# Postoperative external beam irradiation with and without brachytherapy in pelvic node-positive IB1-IIA2 cervical cancer patients: a retrospective clinical study

**DOI:** 10.1186/s13014-015-0495-4

**Published:** 2015-09-17

**Authors:** Lei Li, XinXin Kou, XiaoJie Feng, MingChuan Zhang, HongTu Chao, LiYing Wang

**Affiliations:** Department of Gynecologic Oncology, The Affiliated Tumor Hospital of Zhengzhou University, Zhengzhou, 450008 China

## Abstract

**Background:**

This study assessed the survival outcomes and recurrent patterns in pelvic node-positive IB1-IIA2 cervical cancer patients treated with postoperative external beam irradiation with or without vaginal brachytherapy.

**Methods:**

The records of 1149 cervical cancer patients received radical surgery between February 2008 and March 2010 were retrospectively reviewed. 126 stages IB1-IIA2 patients with positive pelvic lymph node (LN) were included and a total of 113 patients who received different postoperative radiation therapy were identified and analyzed. Of the enrolled patients, 55 patients received pelvic external beam radiotherapy (EBRT) without vaginal brachytherapy and 58 patients received pelvic EBRT with vaginal brachytherapy. Treatment-related toxicities were evaluated. Progression-free survival (PFS) and overall survival (OS) were analyzed using Kaplan-Meier estimates and statistical significance was determined using the log-rank test.

**Results:**

With a median follow-up of 47 months (range: 10–61 months), the group which had pelvic EBRT with brachytherapy had a significantly improved 5-year PFS rate (*P* = 0.044), but no significant difference in 5-year overall survival was found between the two groups (*P* = 0.437). In patients treated without brachytherapy, the most common site of relapse was the pelvis. No significant differences were found regards to acute and chronic radiation toxicities, including myelosuppression, dermatitis, enterocolitis, proctitis and cystitis (*P* = 0.485, 0.875, 0.671, 0.459 and 0.969 respectively) between the groups of pelvic EBRT with and without vaginal brachytherapy.

**Conclusions:**

Treated with pelvic EBRT in combination with vaginal brachytherapy, cervical cancer patients with positive pelvic lymph node had a reduced risk of locoregional recurrence without increased side effects compared with patients treated with pelvic EBRT without vaginal brachytherapy.

## Background

Cervical cancer remains one of the most common malignancies in women worldwide and is also a prominent cause of female cancer death. Treatment for early-stage disease involves radical hysterectomy (RH) with pelvic and/or para-aortic lymph node (LN) dissection or radiation therapy (RT) [1], the former of which is used for most patients because it has the advantage of removing the primary disease and curing the patient within the shortest time period possible. Moreover, RH with pelvic and/or para-aortic LN dissection allows for accurate surgical staging and more accurately targeted adjuvant therapy [2]. Following surgery, there are some surgicopathologic factors that predict disease recurrence. These factors include primary tumor diameter, depth of stromal invasion, lymph-vascular space invasion, presence or absence of parametrial extension, lymph node involvement, and status of resection margins [3–5]. Patients with LN metastasis are considered at high risk for recurrence and should receive postoperative adjuvant therapy [6, 7], such as pelvic external-beam radiation therapy with or without vaginal brachytherapy [8, 9]. Several studies have found that postoperative concurrent chemoradiotherapy can improve the survival in early-stage cervical cancer patients with adverse risk factors by comparing with pelvic radiation therapy alone as adjuvant therapy [6, 10]. Few studies, however, have evaluated the outcomes of different adjuvant therapy strategies for postoperative node-positive patients with or without vaginal brachytherapy, and standard postoperative adjuvant therapy has not been established [11, 12]. Therefore, the aim of this study was to evaluate the long-term outcomes of cervical carcinoma patients with positive pelvic lymph nodes underwent pelvic three-dimensional conformal radiotherapy (3D-CRT) with or without vaginal brachytherapy and treatment-related toxicities, as these patients may benefit from these treatments with improved survival.

## Materials and methods

### Patients

**Fig. 1:**
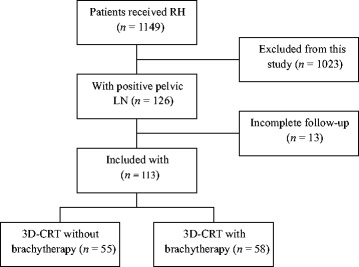
Flow chart showing study numbers and reasons for exclusion in study. The numbers of patients are indicated in parentheses

The Institutional Review Board of the Tumor Hospital of Zhengzhou University reviewed and approved this study (15CT062) and all study procedures conducted according to the principles expressed in the Declaration of Helsinki. A search was performed to identify cervical cancer patients with FIGO stage IB1-IIA2 disease without evidence of distant disease who were treated between February 2008 and March 2010 by radical abdominal hysterectomy and lymphadenectomy with/without bilateral salpingo-oophorectomy (Fig. 1). A total of 1149 cervical cancer patients were identified and 1005 patients with negative pelvic LN and 17 patients with positive para-aortic LN found by surgical staging or by CT-based scan before pelvic external-beam RT were excluded in this study and 126 patients met those criteria finally. Thirteen patients had incomplete follow-up and were not included in the study. Of the included 113 patients, 55 patients received pelvic 3D-CRT and concurrent chemotherapy without vaginal brachytherapy (3D-CRT without brachytherapy group), and 58 patients were treated with 3D-CRT and concurrent chemotherapy with a vaginal brachytherapy boost (3D-CRT with brachytherapy group). Patients with any component of serous neuroendocrine or clear cell differentiation within the tumor and patients with other high risk factors, including parametrial extension and positive margins, were excluded from the analyses. The tumor status and general medical problems of the patients were recorded from their medical records. Informed consent was signed by all patients before treatment.

### External beam RT

Postoperative radiotherapy was performed within 4 weeks of radical surgery. The pelvic radiotherapy consisted of a 6 M-volt (MV) X-ray delivered by a linear accelerator using the anteroposterior parallel opposing technique. Fifty-five patients who underwent pelvic but not para-aortic node dissections received whole pelvis irradiation with 3D-CRT. The median radiation dose was 50 Gy, and it was delivered to areas at risk for microscopic disease in 1.8 or 2.0 Gy fractions once daily for 5 days per week. The pelvic treatment fields generally extended superiorly to include L5. When lateral fields were used, the posterior border encompassed S2. The clinical target volume (CTV) was defined as the area of potential microscopic disease and included the supravaginal portion, cervical stump, paracervical tissue, common iliac lymph nodes, internal and external iliac lymph nodes, obturator lymph nodes, and sacral lymph nodes.

### Intracavitary RT

Fifty-eight patients also received vaginal brachytherapy weekly during external RT. The prescribed dose was 4–7 Gy per fraction to the proximal 3–4 cm of vaginal cuff, with a total dose of 10–15 Gy administered in 1–3 fractions with the integrated after loading treatment planning system. The doses were delivered using high-dose-rate source iridium-192 with intracavitary vaginal ovoid applicators. During the treatment, patients were evaluated weekly by pelvic examination, and complete blood counts and renal and liver function tests were also performed weekly.

### Concurrent chemotherapy

Patients treated with RT in both groups also received platinum-based chemotherapy, which consisted of 40 mg/m^2^ cisplatin weekly.

### Follow-up evaluation

After the completion of treatment, patients were evaluated every 3 months after completion of treatment for the first year, every 6 months during the following 2 years, and annually thereafter. At each visit, bimanual examination, physical examination, and vaginal cytology were performed for the detection of loco-regional recurrence. Ultrasound or CT scans of the abdomen and pelvic region were performed. Suspected cases of recurrent disease were confirmed by biopsy whenever possible. Progression-free survival (PFS) was calculated from the date of radiotherapy initiation to the date of occurrence of disease progression or the date of last follow-up. Overall survival (OS) was calculated from the date of diagnosis to the date of death or, for surviving patients, to the date of last follow-up. The cause of deaths due to disease, directly or indirectly from treatment-related complications, and unknown causes that occurred less than five years after treatment was confirmed by correspondence, telephone or medical record review. Surviving patients were censored on the date of last follow-up. Sites of recurrence were classified as pelvic or extrapelvic. Pelvic recurrence was further classified as recurrence in the central or peripheral pelvic wall. Extrapelvic sites of recurrence included the paraaortic lymph nodes and other sites, such as the supraclavicular lymph nodes and viscera.

### Statistical analysis

OS and PFS were calculated using the Kaplan-Meier method. Differences between groups were analyzed using the log-rank statistic. The clinical characteristics of the patients, local control, survival, toxicities, and the dosimetric parameter were considered categorical variables and were compared between the two groups using the Student *t* test and *X*^2^ test. Statistical significance was defined at a level of *P* < 0.05. All analyses were performed using SPSS version 17.0 (SPSS Inc., Chicago, IL).

## Results

### Patient and tumor characteristics

Amount the 1149 patients who received Piver type III radical hysterectomy and lymphadenectomy with/without bilateral salpingo-oophorectomy, 126 patients (10.9 %) were found had pelvic lymph note metastasis. The demographics and clinical characteristics of the patients in this study are summarized in Table 1. The median age of the patients in 3D-CRT without brachytherapy group was 49 years (range 26–73) and the median age of the patients in the 3D-CRT with brachytherapy group was 50 years (range 27–76). There were no statistically significant differences in terms of age, tumor stage, histology, grade, number of dissected lymph nodes and number of positive lymph nodes.

**Table 1:** Clinical and pathologic characteristics of patients by treatment group

	3D-CRT without brachytherapy (*n* = 55)	3D-CRT with brachytherapy (*n* = 58)	*P* value
Age (years, median)	49 (26–73)	50 (27–76)	0.313
FIGO stage			0.947
IB1	11 (20.0 %)	9 (15.5 %)	
IB2	13 (24.6 %)	15 (25.9 %)	
IIA1	19 (34.5 %)	24 (41.4 %	
IIA2	12 (21.8 %)	10 (17.2 %)	
Histology			0.785
Squamous cell carcinoma	47 (85.5 %)	51 (87.9 %)	
Adenocarcinoma	8 (14.5 %)	7 (12.1 %)	
Grade			0.719
1	8 (14.5 %)	9 (15.5 %)	
2	30 (54.4 %)	33 (56.9 %)	
3	17 (30.9 %)	16 (27.6 %)	
Number of retrieved lymph nodes	24.9 ± 4.2	24.7 ± 3.7	0.825
Number of positive lymph nodes			1
≤3	51 (92.7 %)	53 (96.4 %)	
>3	4 (7.3 %)	5 (3.6 %)	

### Clinical outcomes

The median follow-up for patients was 47 months (range: 10–61 months). All time intervals were measured from the time of diagnosis. In the 3D-CRT without brachytherapy group, at the time of the last follow-up, 41 of 55 patients were alive without evidence of disease, 14 patients had experienced recurrence, and nine patients had died. Of the 14 patients with recurrence, ten patients had pelvic recurrence, including six patients with vaginal recurrence, four patients failed with pelvic recurrence, one with para-aortic region recurrence and three patients had distant failure. In 3D-CRT with brachytherapy group, at the time of the last follow-up, 52 of 58 patients were alive without evidence of disease and six patients had experienced recurrence. Of the patients with recurrent, five patients experienced distant failure and died from cervical cancer, and only one patient experienced peripheral pelvic wall recurrence.

**Fig. 2:**
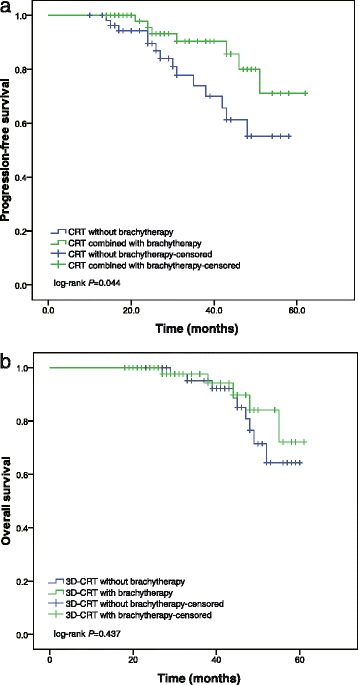
Kaplan-Meier survival curves of progression free survival (PFS) and overall survival (OS): (a) PFS; (b) OS

A statistically significant difference in PFS was observed between the different adjuvant treatment groups. The 5-year PFS rate was higher in patients treated with 3D-CRT with brachytherapy than in those treated with 3D-CRT only; the PFS at 5 years was 71.1 % in the combined-therapy group and 55.1 % in the 3D-CRT alone group according to the Kaplan-Meier analysis, and this difference was statistically significant (*P* = 0.044) (Fig. 2a). The 5-year OS rate for the patients treated with and without vaginal brachytherapy were 72.1 and 64.3 %, respectively, and this difference was not statistically significant (*P* = 0.437) (Fig. 2b).

### Acute and chronic radiation toxicities

No treatment-related deaths were observed. Acute and chronic toxicities were assessed using the Acute Radiation Morbidity Scoring Criteria and the Late Radiation Morbidity Scoring Scheme of the RTOG. Toxicities that occurred more than 3 months after the start of radiotherapy were considered as late toxicities. The major acute reactions observed included myelosuppression, proctitis, dermatitis bellyache, diarrhea, and acute cystitis. Myelosuppression was the most common undesirable side effect for both groups. No significant differences were found in leukopenia, dermatitis, enterocolitis, proctitis and cystitis between the two groups (*P* = 0.485, 0.875, 0.671, 0.459 and 0.969 respectively), as shown in Table 2.

**Table 2:** Incidences of acute and late toxicities in groups given 3D-CRT with/without brachytherapy groups according to RTOG/EORTC criteria

Grade	3D-CRT without brachytherapy (*n* = 55)	3D-CRT with brachytherapy (*n* = 58)	*P* value
Acute side effect			
Leukopenia			0.485
0	6 (10.9 %)	9 (15.5 %)	
1	15 (27.3 %)	17 (29.3 %)	
2	22 (40.0 %)	20 (34.5 %)	
3	9 (16.4 %)	10 (17.2 %)	
4	3 (5.5 %)	2 (3.4 %)	
Dermatitis			0.875
0	31 (56.4 %)	34 (58.6	
1	20 (36.4 %)	19 (32.8 %)	
2	4 (7.3 %)	5 (8.6 %)	
3	0 (0 %)	0 (0 %)	
4	0 (0 %)	0 (0 %)	
Enterocolitis			0.671
0	32 (58.2 %)	33 (56.9 %)	
1	20 (36.4 %)	18 (31.0 %)	
2	3 (5.5 %)	6 (10.3 %)	
3	0 (0 %)	1 (1.7 %)	
4	0 (0 %)	0 (0 %)	
Late side effect			
Proctitis			0.459
0	30 (54.5 %)	29 (50.0 %)	
1	18 (32.7 %)	23 (39.7 %)	
2	7 (12.7 %)	8 (13.8 %)	
3	0 (0 %)	1 (1.7 %)	
4	0 (0 %)	0 (0 %)	
Cystitis			0.969
0	34 (61.8 %)	35 (60.3 %)	
1	14 (25.5 %)	15 (25.9 %)	
2	7 (12.7 %)	6 (10.3 %)	
3	1 (1.8 %)	2 (3.4 %)	
4	0 (%)	0 (0 %)	

## Discussion

Women with cervical cancer detected at the early stage are often treated with radical hysterectomy (RH) combined with pelvic and/or para-aortic lymph node (LN) dissection. After surgery, several prognostic factors indicate the need for additional treatment, including parametrial involvement, vaginal invasion, positive pelvic lymph nodes, lymph-vascular-space invasion, deep stromal invasion, and tumor size, and the former three are defined as high risk factors that necessitate adjuvant radiotherapy [3–5]. The presence of positive pelvic lymph nodes was the most frequently encountered high risk factor, and it is always considered an independent prognostic factor [6–9]. Uno et al. found that the 5-year OS rates for patients with and without pelvic lymph node metastasis were 52 and 89 %, respectively, and the relapse-free survival rates for those patients were 44 and 83 %, respectively [13]. Stock RG et al. reported that patients who received postoperative whole pelvic irradiation had significantly improved pelvic control, disease-free survival, and overall survival rates [8]. However, most patients with LN metastasis received adjuvant postoperative radiotherapy and platinum-based chemotherapy without vaginal brachytherapy, and intracavitary radiotherapy was only combined with external-beam radiotherapy at some institutions and only in cases with involved vaginal resection margins [14, 15]. Few studies have assessed the postoperative survival outcomes of patients with pelvic lymph node metastasis with/without brachytherapy. Therefore, the current study investigated the impact of vaginal brachytherapy in patients with pelvic lymph node metastasis after RH and attempted to determine whether brachytherapy was necessary in these patients [16, 17] .

In this retrospective study, we compared the DFS of patients with pelvic lymph node metastasis who received pelvic external beam radiotherapy with brachytherapy postoperatively to those of patients who received pelvic EBRT alone. The Kaplan-Meier survival analysis demonstrated that PFS differed significantly between these two groups. The 5-year progression-free survival rate was higher in patients treated with 3D-CRT combined with vaginal brachytherapy than in those treated with pelvic EBRT alone. Patient prognosis and the pattern of treatment failure correlated with the method of treatment. Patients in the combined group had less local progression than those in the group given pelvic EBRT alone. For patients who received pelvic RT only, the most common first site of relapse was the pelvis; in contrast, only one of the patients treated with external beam RT plus brachytherapy experienced pelvic recurrence, suggesting that pelvic EBRT alone is not adequate to prevent local recurrence.

Intracavitary brachytherapy involves the application of a radioactive source in close proximity to the upper part of vaginal cuff, which allows for a high dose to the tumor bed with relative sparing of the surrounding normal structures and are often given after EBRT is completed. As Kim et al. found that among patients who had received postoperative radiotherapy, 41 % failed in the pelvis, and mainly at vaginal wall and vaginal cuff, which partly explains the improved PFS at combined treatment group in the current study [18]. The benefit of vaginal cuff brachytherapy can also be found in other type of gynecological cancer, for example, the results of Low’s study found that adjuvant chemotherapy, EBRT, and vaginal brachytherapy for high risk endometrial cancer patients demonstrated 15 % pelvic recurrence and 0 % vaginal recurrence [19]. The reduction in local recurrence with the addition of brachytherapy will benefit a larger number of patients at high-risk and will result is a higher DFS rate [7, 20].

To maximize PFS while minimizing treatment-related morbidity, it is important to determine the risks and benefits of adjuvant RT and chemotherapy after radical surgery for each patient individually. Although complications are often underreported in retrospective reports, the majority of patients are able to finish the prescribed radiation regimen, including both external beam radiation and brachytherapy, and most patients undergo radiation therapy with minimal side effects. The rates of acute and late complications in our study were acceptable and comparable to those of previous reports, such as a report by Sedlis of severe and life-threatening toxicities in 6 % of irradiated patients [21]. Moreover, side effects at bladder or rectum with the addition of brachytherapy were not significantly to the recorded doses. These results indicate that the combination of adjuvant therapy with brachytherapy was well-tolerated and that this treatment regimen should be recommended as a more effective method for postoperative adjuvant RT in node-positive cervical cancer patients. However, the reported rate of brachytherapy use was lower than expected, as the equipment and number of staff were insufficient to perform brachytherapy in some gynecologic oncology departments, especially in small non-academic institutions [22, 23].

In conclusion, we observed that the combination of pelvic EBRT and concomitant chemotherapy with vaginal brachytherapy was more effective for the treatment of pelvic node-positive cervical cancer than pelvic EBRT and concomitant chemotherapy alone. The inclusion of brachytherapy reduced the local recurrence rate of cervical cancer, which led to a higher disease-free survival rate, and did not increase the rates of side effects, such as hematologic toxicity. Additionally, the incidence of late side effects was similar between the two treatment groups. This preliminary study suggests that pelvic EBRT with vaginal brachytherapy is feasible and may have significant clinical implications for cervical cancer treatment; therefore, brachytherapy should be considered in future treatment strategies for patients who are candidates for post-irradiation RT after surgical treatment.

The limitations of our study include the methodological uncertainties of the diagnostic procedures and the relatively low number of patients available for assessment. There are also several limitations in this study. Firstly, different types of radical hysterectomy were not compared, and para-aortic LN dissection was not performed routinely, which is also a prognostic factor for PFS and OS. Secondly, no concrete treatment policy about addition of brachytherapy has been build, which make the adding of brachytherapy depending the discretion of the gynecologic oncologist. Moreover, due to the retrospective design of this study, recall bias is likely to exist. Therefore future prospective clinical trials are needed to validate the results of the current study and to evaluate the most effective RT doses for the treatment of cervical cancer patients.
